# Digitale Lehre 2020: Studenten schätzen die Aufmerksamkeit während einer Onlinevorlesung gleichwertig zu einer Präsenzvorlesung ein

**DOI:** 10.1007/s00347-021-01344-1

**Published:** 2021-03-02

**Authors:** Armin Mohi, Stefanie Gniesmer, Mahdy Ranjbar, Vinodh Kakkassery, Swaantje Grisanti, Birte Neppert, Maximilian Kurz, Julia Lüke, Matthias Lüke, Maya Müller, Claudia Lommatzsch, Salvatore Grisanti

**Affiliations:** 1 Klinik für Augenheilkunde UKSH, Universität zu Lübeck, Ratzeburger Allee 160, Lübeck, Deutschland; 2 Augenheilkunde am Rheincenter, Köln, Deutschland; 3 Institut für Refraktive- und Ophthalmo-Chirurgie (IROC), Zürich, Schweiz; 4 Augenzentrum am St. Franziskus-Hospital Münster, Münster, Deutschland

**Keywords:** Digitale Lehre, Digitalisierung, Hochschuldidaktik, COVID-19, Informationstechnologie, Digital teaching, Digitalization, University didactics, COVID-19, Information technology

## Abstract

**Hintergrund:**

Die Corona-Pandemie hat zu einer kurzfristigen Anpassung der Lehrveranstaltungen im Studiengang Humanmedizin geführt. Präsenzlehre war in vielen Bereichen nicht mehr möglich, sodass ein digitales Lehrkonzept etabliert werden musste, um weiterhin eine adäquate medizinische Ausbildung zu gewährleisten.

**Methodik:**

Basierend auf den Lehrinhalten unserer Präsenzveranstaltung, haben wir ein digitales Curriculum erarbeitet. Primäre Werkzeuge zur Umsetzung dieses Vorhabens waren Cisco WebEx und Moodle, welche bereits an der Universität zu Lübeck etablierte Softwarelösungen waren. Anschließend wurde anhand einer Umfrage unter den Studierenden dieses Konzept evaluiert.

**Ergebnisse:**

Die Auswertung der Evaluation hat gezeigt, dass Inhalt und Didaktik der Lehrveranstaltung als „gut bis sehr gut“ bewertet wurden. Die Kommunikation mit den Studenten und unter den Studenten selbst wurde als „gut“ eingestuft. Es hat sich insbesondere gezeigt, dass die Aufmerksamkeit der Studenten während der Vorlesung als „gleichwertig“ zu der Aufmerksamkeit einer Präsenzvorlesung bewertet wurde. Die Vermittlung von praktischen Fähigkeiten wurde erwartungsgemäß als „schlecht“ beurteilt. Abschließend konnte sich ein Großteil der Studierenden eine Kombination aus digitaler und Präsenzlehre auch in Zukunft vorstellen.

**Diskussion:**

Die kurzfristige Umstellung auf einen digitalen Lehrbetrieb hat Lehrende und Studierende vor eine Herausforderung gestellt. Durch geeignete Softwarelösungen können theoretische Inhalte adäquat vermittelt werden. Die Studenten werteten die digitale Lehrveranstaltung im Vergleich zur Präsenzlehre als gleichwertig und auch für die Zukunft als eine attraktive Option. Ein weiterhin zu lösendes Problem bleibt aber das Erlernen von praktischen Fähigkeiten.

**Video online:**

Die Online-Version dieses Beitrags (10.1007/s00347-021-01344-1) enthält (ein) Video(/s).

## Abstract (en)

**Background:**

The corona pandemic has led to short-term adjustments of the procedures in medical courses. In many departments classroom teaching was no longer possible so that it was necessary to implement digital courses to provide an adequate medical training.

**Methods:**

We developed a digital curriculum based on the contents of our established courses using primary in-house tools, such as Cisco WebEx and Moodle. We then performed a student survey to evaluate the implementation of the concept.

**Results:**

The evaluation showed good ratings according to teaching content and didactics. The communication with the students and between each other was also considered good. Students assessed attention during the course to be equivalent in comparison to classroom teaching. As expected, the training of practical skills was evaluated as poor. For future courses most of the students could imagine a combination of digital and classroom teaching.

**Discussion:**

Implementing a digital course was a sudden challenge for students and tutors. With suitable software solutions and good communication, it is possible to adequately teach theoretical content via digital courses. Students assessed the digital training in most parts to be a good and an equivalent option compared to classroom teaching. A remaining problem is the lack of practical training.

Die digitale Lehre hat durch die Corona-Pandemie in nahezu allen Fachdisziplinen einen Aufschwung erlebt. Während unser Alltag zunehmend von digitalen Medien geprägt ist, war die universitäre Lehre bisher in der Medizin noch größtenteils analog. Lehrende und Studierende an allen Fakultäten mussten sich kurzfristig auf diese Situation einstellen. In diesem Beitrag wollen wir darstellen, wie wir an der Augenklinik der Universität zu Lübeck diese Herausforderung angegangen sind und wie unsere Umsetzung der digitalen Lehre von den Studierenden wahrgenommen wurde.

## Hintergrund

Die Corona-Pandemie hat die Gesellschaft in allen Bereichen unerwartet getroffen und einen Großteil des Alltags verändert. Auch die curriculare Lehre musste kurzfristig auf diese Situation reagieren, um die universitäre Ausbildung wenigstens in Grundzügen zu gewährleisten.

Mit dem Erlass der Landesregierung Schleswig-Holsteins vom 12.03.2020 kam es zum Verbot von öffentlichen Veranstaltungen an Hochschulen nach § 28 des Infektionsschutzgesetzes [1].

Dadurch konnte eine Präsenzlehre in gewohnter Form im Sommersemester 2020 nicht stattfinden. Wir entschieden uns deshalb, das gesamte augenheilkundliche Lehrangebot online durchzuführen.

Die Herausforderung bestand in der kurzfristigen Umsetzung und Umstellung für Studierende und Lehrende, die bis dahin keine Erfahrung mit einem solchen Format hatten.

In diesem Artikel soll dargestellt werden, wie die digitale Lehre in der Augenheilkunde an der Universität zu Lübeck im Sommersemester 2020 angepasst werden musste und wie diese von Studierenden wahrgenommen wurde.

## Material und Methodik

### Aufbau der Präsenzlehre vor der Pandemie

Die Lehrveranstaltung der Augenheilkunde an der Universität zu Lübeck umfasst die Hauptvorlesung sowie das Blockpraktikum. Am Ende des Semesters erfolgt eine schriftliche Prüfung zur Überprüfung des Lernerfolgs.

Die wöchentliche Hauptvorlesung (90 min) umfasst grob die gesamte Augenheilkunde und betont die Bereiche, die interdisziplinär relevant sind.

Das in 4 Blöcke aufgeteilte Praktikum beinhaltet die Schwerpunkte „Netzhaut“, „Notfälle“, „Neuroophthalmologie“ und „Orthoptik“. Jeder Block besteht aus einer 60-minütigen Kursvorlesung, gefolgt von 45 min für praktische Übungen (Skills-Lab, Nahtkurs, Hospitation im Operationssaal, Orthoptik) und abschließendem fallbasiertem Repetitorium.

### Umsetzung der digitalen Lehre während der Pandemie

Aus organisatorischen Gründen und um für die Lehrenden eine partielle Kontinuität zu gewährleisten, entschieden wir uns, den strukturellen Aufbau der Lehre nicht zu verändern. Der Wegfall der praktischen Übungen konnte jedoch nicht adäquat digital kompensiert werden. Hierfür wurden zusätzliche Klinikhospitationen während der vorlesungsfreien Zeit angeboten, um in kleinerem Rahmen die wichtigen praktischen Fähigkeiten zu vermitteln.

Als Grundlage für die digitale Lehre haben wir uns der Videokonferenzplattform WebEx meetings (Cisco Systems, San José, CA, USA) bedient. Zur Organisation der Lehre wurde das bereits etablierte Kursmanagementsystem Moodle (Opensource, moodle.org, West Perth, Australien) genutzt.

Die Abschlussklausur wurde ebenfalls online in Moodle durchgeführt.

### Vorlesungen

Die Vorlesungen wurden zu der gewohnten Zeit als WebEx-Seminar abgehalten. Das Meeting konnte nur mit einem eigenen Link betreten werden. Diesen Link haben die Studierenden einen Tag vor der Vorlesung per „Ankündigung“ in Moodle und damit auch automatisch per E‑Mail erhalten. Der Dozent wurde ebenfalls zu dem Meeting eingeladen und als Moderator festgelegt. Es wurde entschieden, die Vorlesung live in WebEx zu halten und zeitgleich aufzuzeichnen. Die Aufzeichnung erfolgte abschnittsweise entsprechend den Themenblöcken der jeweiligen Vorlesung und wurde in der WebEx-Cloud abgespeichert. Anschließend wurde der Link zu diesen Videos über Moodle freigeben.

**Figure 1:**
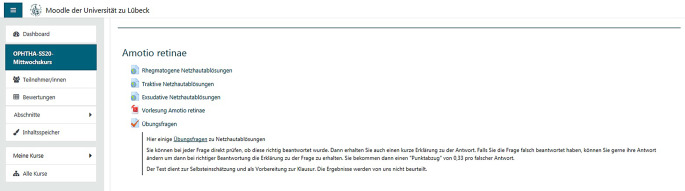

Das Aufzeichnen der Vorlesung sollte einerseits die Möglichkeit bieten, dass die Studenten den Unterrichtsstoff wiederholen, zum anderen sollte es den Studenten, die nicht an dem Meeting teilgenommen haben, ermöglichen, die Inhalte in adäquater Form vermittelt zu bekommen (Abb. 1). Ein exemplarisches Video kann unter https://uni-luebeck.webex.com/uni-luebeck/ldr.php?RCID=4aadb4df835e76c5b529c255a88d3168 abgerufen werden (Passwort Ophtha#2020).

Da die Aufmerksamkeitsspanne im Verlauf eines Lehrvideos abnehmen kann, entschieden wir uns für die Segmentierung der Aufzeichnung. Hierdurch konnte eine Screencast-ähnliche Struktur geschaffen werden. Diese ermöglicht es, einzelne Themengebiete auch später wiederholt anzuschauen. Eine optimale Länge pro Themenblock sollte ca. 10–15 min umfassen [3]. Die Aufzeichnungen konnten von den Studenten nur angesehen werden, wenn eine „Einwilligung der Nichtweitergabe von Unterrichtsmaterial“ abgegeben und das richtige Passwort eingegeben wurde. Zudem konnten die Studenten das Video nicht downloaden. So sollte die unkontrollierte Weiterverbreitung des Unterrichtsmaterials eingedämmt werden.

Zur weiteren Unterstützung unterschiedlicher Lernverhalten wurde die Vorlesung zusätzlich als PDF-Datei zur Verfügung gestellt.

### Blockpraktika

Die Blockpraktika wurden ebenfalls als WebEx-Meeting abgehalten. Die in den letzten Jahren etablierte Themenstruktur wurde beibehalten. Auch hier haben die Studenten und Dozenten am Tag vor dem Seminar die Einwahldaten und nötigen Informationen über Moodle per E‑Mail erhalten. Da der praktische Teil ausgefallen ist, wurde in den Seminaren ein Hauptaugenmerk auf Untersuchungstechniken gelegt. Zudem wurden Untersuchungsvideos und auch Operationsvideos gezeigt, um das Seminar anschaulicher zu gestalten. Im Anschluss an die Videos wurde noch ein fallbasiertes „Repetitorium“ gehalten, um Gelerntes direkt anzuwenden.

### Klausur

Die Klausur wurde aufgrund der Hygienemaßnahmen und der daraus resultierenden Raumknappheit ebenfalls online in Moodle absolviert. Hier stellte sich die besondere Herausforderung, dass die Studenten im heimischen Umfeld natürlich die Möglichkeit haben, Informationen aus Büchern oder dem Internet zur Lösung der Fragen heranzuziehen oder sich telefonisch abzusprechen. Daher wurde die Klausur in einem engen Zeitfenster angeboten (75 min für 30 Fragen). Zudem war bei jedem Studenten die Reihenfolge der Fragen komplett vermischt. Es wurden immer 3 Fragen auf einmal zur Beantwortung angeboten. Wenn diese als vom Studenten „beantwortet“ gewertet wurden, konnten diese Antworten später nicht mehr geändert werden. Es wurden Multiple-Choice-Fragen gestellt, teilweise auch mit Bildern.

**Figure 2:**
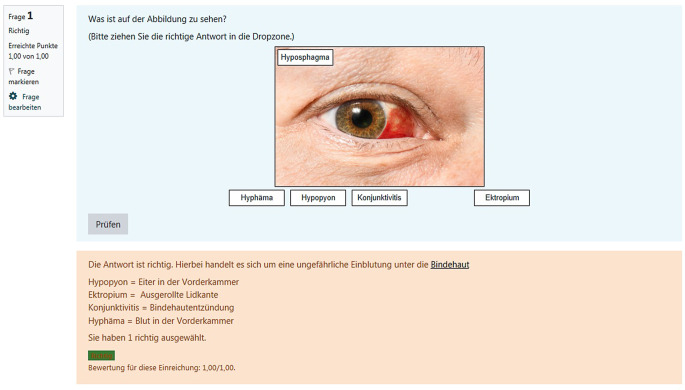

Als Vorbereitung für die Klausur wurden in Moodle zudem Übungsfragen zu den jeweiligen Themenblöcken gestellt. Dort wurden, ähnlich wie einem Lernmodul, auch weitere Informationen zu der Frage und den jeweiligen Antwortmöglichkeiten gegeben, sodass die Studierenden dort nicht nur ihr Wissen überprüfen konnten, sondern auch die Möglichkeit hatten, mit diesen Testfragen zu lernen (Abb. 2).

### Auswertung

Zur Überprüfung der Qualität unserer Lehrveranstaltung haben wir die Studenten gebeten, nach dem Semester an einer speziellen Lehrevaluation teilzunehmen. Die Evaluation bestand aus einem Fragebogen mit 19 Fragen und sollte den Inhalt der Lehrveranstaltung abfragen sowie deren digitale Umsetzung. Die Beurteilung erfolgte größtenteils nach dem Schulnotensystem (Note 1–5) oder nach einer angepassten Likert-Skala (trifft zu (1), trifft eher zu (2), teils-teils (3), trifft eher nicht zu (4), trifft nicht zu (5)).

Der Fragenkatalog wurde zur besseren Auswertung in folgende Themen subgruppiert:

- Inhalt, Didaktik,
- Interaktion,
- Kommunikation,
- technische Umsetzung,
- Vergleich zur Präsenzlehre,
- praktische Fähigkeiten,
- Ausblick.

Zudem wurde die Anzahl der an der Vorlesung eingeloggten Studenten mit der Anzahl der Studenten verglichen, welche im letzten Präsenzsemester (Wintersemester 2019/2020) an der Vorlesung teilgenommen haben (Rücklauf der Feedbackbögen nach jeder Vorlesung).

## Ergebnisse

### Teilnahme

Die Teilnahme an der Vorlesung im Sommersemester 2020 war im Mittel fast doppelt so hoch wie im Wintersemester 2019/2020 (21,7 vs. 44,3 Studenten). Der Fragenkatalog mit seinen 19 Fragen hatte einen Rücklauf von 41 Studenten (Semesterstärke 99 Studenten).

### Fragenkatalog

**Figure 3:**
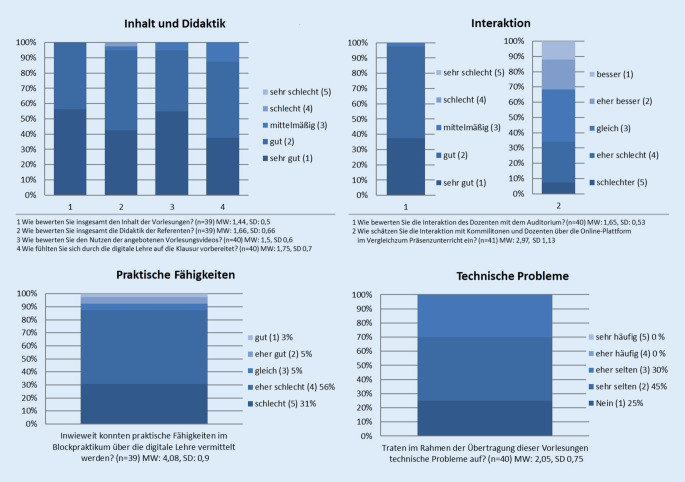

Inhalte und Didaktik der Vorlesung wurden insgesamt als sehr gut beurteilt (Inhalt: Mittelwert [MW] 1,44, Standardabweichung [SD] 0,5; Didaktik: MW 1,65, SD 0,66). Die zusätzlich angebotenen Lehrinhalte in Moodle, wie z. B. Videos, Vorlesungen und Übungsfragen, wurden als gut und nützlich beurteilt (MW 1,5, SD 0,6). Zudem fühlten sich die Studenten durch den Unterricht gut auf die Klausur vorbereitet (MW 1,75, SD 0,7). Auch die Interaktion während der Vorlesung wurde als gut beurteilt (MW 1,65, SD 0,53), genauso wie die Interaktion mit den Dozierenden und den Kommilitonen über die Online-Plattformen (Kommunikation: besser (1), eher besser (2), gleich (3), eher schlechter (4), schlechter (5); MW 2,97, SD 1,13; Abb. 3).

Technische Probleme (Abb. 3) wurden als „sehr selten“ angegeben (technische Probleme: nein (1), sehr selten (2), selten (3), häufig (4), sehr häufig (5); MW 2,05, SD 0,75).

**Figure 4:**
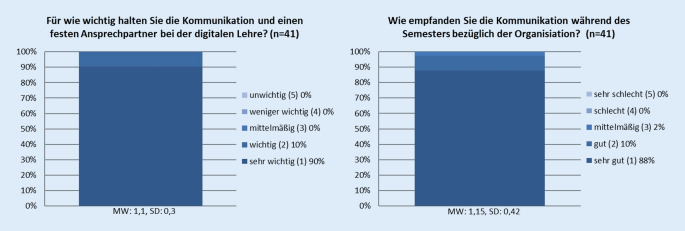

Im Bereich der Kommunikation gaben die befragten Studenten an, dass sie eine gute Kommunikation und einen festen Ansprechpartner während des Semesters als sehr wichtig erachten (MW 1,1, SD 0,3), und gaben uns auch das Feedback, diese Kommunikation über die Moodle-Plattform sehr gut gestaltet zu haben (MW 1,15, SD 0,42) (Abb. 4).

**Figure 5:**
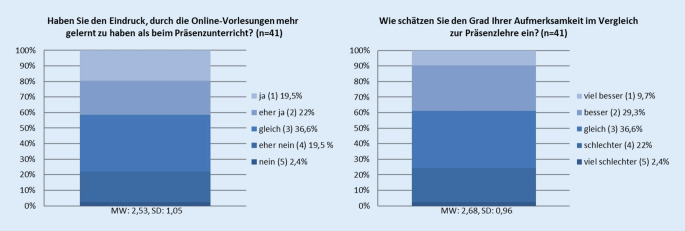

Im Vergleich zur Präsenzlehre gaben die befragten Studenten an, dass sie den Grad der Aufmerksamkeit ähnlich zu einer Präsenzvorlesung einschätzen (Grad der Aufmerksamkeit: viel besser (1), besser (2), gleich (3), schlechter (4), viel schlechter (5); MW 2,68, SD 0,96), und auch, dass sie den Eindruck hatten, einen zur Präsenzveranstaltung ähnlichen Lerngewinn zu haben (Lerngewinn im Vergleich: viel besser (1), besser (2), gleich (3), schlechter (4), viel schlechter (5); MW 2,53, SD 1,05; Abb. 5).

Die Vermittlung von praktischen Fähigkeiten wurde insgesamt als schlecht bewertet (Vermittlung von praktischen Fähigkeiten: gut (1), besser (2), gleich (3), eher schlecht (4), schlecht (5); MW 4,08, SD 0,9; Abb. 3).

**Figure 6:**
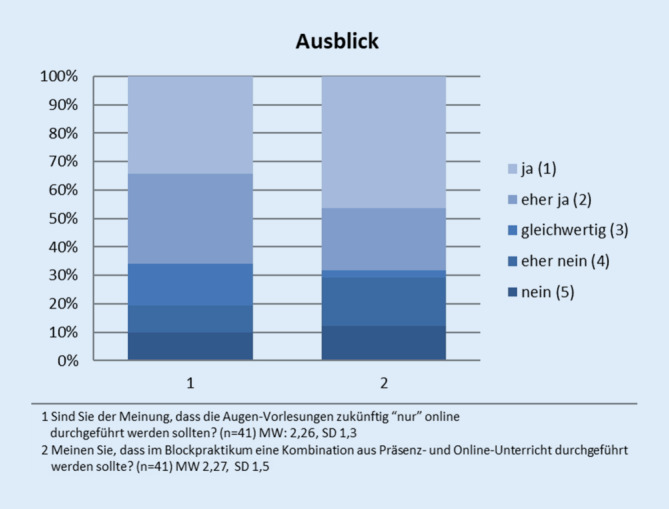

Perspektivisch können sich ca. 2/3 der Studenten vorstellen, dass die Vorlesung Augenheilkunde künftig nur noch online gehalten wird (ja (1), eher ja (2), gleich (3), eher nein (4), nein (5); MW 2,26, SD 1,3) und das Blockpraktikum Augenheilkunde in einer Mischung aus Online- und Präsenzlehre stattfindet (ja (1), eher ja (2), gleich (3), eher nein (4), nein (5); MW 2,27, SD 1,5; Abb. 6).

### Klausur

Die Klausur beinhaltete genau wie im vorherigen Semester 30 Multiple-Choice-Fragen. Wir verzeichneten ein überdurchschnittlich gutes Abschneiden der Studenten in der Klausur. Der Notendurchschnitt der digitalen Klausur lag bei 1,14. Im Vergleich dazu war der Notendurchschnitt der letzten 3 Klausuren im Mittel bei 2,44 (Wintersemester 18/19: 2,4, Sommersemester 2019: 2,4, Wintersemester 19/20: 2,51).

## Diskussion

Die Corona-Pandemie hat uns innerhalb kürzester Zeit vor das Problem gestellt, eine gute Lehre und Medizinerausbildung ohne Präsenz zu ermöglichen und zu gewährleisten. Hierfür waren an unserer Universität etablierte Plattformen wie Moodle und Cisco WebEx sehr hilfreich. Wir sind dabei frühzeitig an die Studenten herangetreten und haben offen die Herausforderungen angesprochen. Kernaussagen unserer Umfrage befassen sich mit dem Thema Kommunikation und dem subjektiven Vergleich zur Präsenzlehre.

Aus unserer Umfrage geht hervor, dass die Studenten in solch einer besonderen Situation eine gute Kommunikation als sehr wichtig einschätzen. Die direkte Kommunikation mit den Studenten über die Moodle-Plattform hat auch den Vorteil, Anregungen und Wünsche der Studierenden zügig anzunehmen und umzusetzen. Hierbei wurden sowohl inhaltliche Themen als auch technische Fragen adressiert.

Es ist interessant zu beobachten, dass die befragten Studenten die Aufmerksamkeit und den Lerngewinn der Onlinevorlesung mit der Präsenzvorlesung gleichsetzen, war doch zu befürchten, dass im heimischen Umfeld am PC die Aufmerksamkeit schnell abdriften könnte. Um dies gezielt abzuwenden, wurde in den Seminaren aktiv auf die Studenten eingegangen. Wie im Präsenzunterricht wurden die Studenten mit häufigen Fragen oder Aufforderungen, bestimmte Abbildungen zu erläutern, aktiv zur Interaktion aufgefordert.

Der Zugang zur Vorlesung aus der heimischen Umgebung ist zudem ungleich einfacher, was auch die überdurchschnittliche Teilnahme an der Vorlesung erklären könnte, insbesondere in Anbetracht der Tatsache, dass die Vorlesungen komplett als Video online zur Verfügung gestellt wurden. Des Weiteren wurde über die Möglichkeit, die Vorlesung als „screencast“ zu nutzen und ein aus Übungsaufgaben bestehendes Lernmodul anzubieten, ein Mehrwert zu der bisherigen konventionellen Lehrveranstaltung geschaffen. Die positive Resonanz bezüglich Vorlesungsvideos als zusätzliches Lehrmaterial und die größtenteils gut funktionierende technische Umsetzung der Onlinelehre deckt sich mit ähnlichen Beobachtungen zu diesem Thema aus diesem Jahr [4].

Während Inhalte und Didaktik einer Vorlesung grundsätzlich auch vom Dozenten abhängig sind, konnten wir über das gesamte Kollegium hindurch gute Werte in diesem Bereich erlangen. Es ist erfreulich zu sehen, dass insbesondere die Interaktion während einer Onlinevorlesung als gut bewertet wurde, sind hier doch gewisse Einschränkungen technischer Natur zu erwarten.

Eine klare Limitation der Onlinelehre stellt das Fehlen der praktischen Übungen dar. Praktische Fähigkeiten haben natürlich einen hohen Stellenwert in der Ausbildung junger Mediziner [5].

Weder durch Untersuchungs- noch Operationsvideos kann dies kompensiert werden. Webbasierte Trainingstools stellen zudem in der Augenheilkunde zum jetzigen Zeitpunkt kaum Alternativen dar. Diese sind zum einen momentan in zu geringem Ausmaß auf dem Markt [2] und zum anderen häufig an Ärzte in der Facharztausbildung gerichtet. Trainingstools, die Untersuchungstechniken simulieren, sind zwar bereits etabliert [6], werden aber derzeit nicht am heimischen PC angewandt. Die Nutzung kann nur an Schulungszentren bzw. in der Klinik erfolgen. Der Einsatz eines solchen Simulators würde es jedoch ermöglichen, zu anderen Schulungsteilnehmern ausreichend Abstand zu halten, da der zu Untersuchende kein Proband oder Kommilitone ist.

Ein interessantes Ergebnis der Befragung zeigt, dass ein Großteil der Studenten sich nach diesem Semester durchaus eine Kombination aus Online- und Präsenzlehre für die Zukunft vorstellen kann. Da wir noch auf unbestimmte Zeit die Auswirkungen der Corona-Pandemie zu bewältigen haben, wurde dieser Aspekt in der Planung des nächsten Semesters aufgenommen. Dort werden wir den Studenten zumindest an einem Termin die Möglichkeit geben können, in Kleinstgruppen praktische Erfahrungen zu sammeln. Zudem gibt uns die gewonnene Erfahrung auf diesem Gebiet auch die Möglichkeit, in den Zeiten nach der Pandemie gezielt Lehrinhalte online zu vermitteln. Der Zusatznutzen, welcher z. B. durch eine aufgezeichnete Vorlesung entsteht, wurde von unseren Studenten als sehr gut beurteilt und kann auch in Zukunft dabei helfen, eine attraktive Lehrveranstaltung anzubieten.

Im Bereich der Leistungsüberprüfung ist dagegen eine Onlineklausur nach unseren Erfahrungen diskussionswürdig. Das sehr gute Abschneiden der Studenten wird weniger mit der „digitalen Vorbereitung“ auf die Klausur zu erklären sein als durch die Möglichkeit, während einer digitalen Klausur Wissenslücken sehr kurzfristig auszugleichen. Hier muss aber auch klargestellt werden, dass in solch einer besonderen Situation diese Problematik zugunsten der Studenten in Kauf genommen wurde.

## Fazit für die Praxis

- Durch digitale Lehrformate können theoretische Inhalte adäquat vermittelt werden.
- In unserem Setting wurden der Lerngewinn und die Aufmerksamkeit während der Vorlesung als gleichwertig zu einer Präsenzveranstaltung eingeschätzt.
- In Zukunft kann die gesammelte Erfahrung in diesem Gebiet genutzt werden, um durch digitale Lehrinhalte die Präsenzlehre gezielt aufzuwerten.

## Supplementary Information

Beispielvideo
